# Physicochemical and sensory characteristics of commercial, frozen, dry, and wet-aged Hanwoo sirloins

**DOI:** 10.5713/ajas.18.0610

**Published:** 2019-03-07

**Authors:** Ji-Han Kim, Min-Young Jeon, Chi-Ho Lee

**Affiliations:** 1 Department of Food Science and Bioproduct Sciences, University of Saskatchewan, Saskatoon, SK, S7N5A8, Canada; 2 Department of Food Science and Biotechnology of Animal Resources, Konkuk University, Seoul 05029, Korea

**Keywords:** Wet-aging, Dry-aging, Hanwoo, Frozen Meat, Electronic Tongue

## Abstract

**Objective:**

The objective of this study was to evaluate the physicochemical, sensory and taste characteristics of commercial, frozen, dry, and wet aged Hanwoo sirloin.

**Methods:**

Grade 2 sirloin from 6 Hanwoo steers (about 30 months old) were obtained after 5 days *postmortem*. Samples were assigned to four groups which were commercial beef (CON, control group), frozen beef (Hanwoo frozen, HF; 40 days in −18°C freezer), wet-aged beef (Hanwoo wet-aging, HW; 21 days), and dry-aged beef (Hanwoo dry-aging, HD; 40 days). HW and HD were stored in a 80%±5% relative humidity cooler at 1°C.

**Results:**

The HF group showed a significantly higher cooking loss and expressible drip with significantly higher pH compared to other groups. In addition, protein and fat contents in the HD group were higher than those in other groups (p<0.05). The shear forces in the HW and HD groups were significantly lower than those in the CON group. The HD group had significantly higher omega-3 and polyunsaturated fatty acids compared with other groups. Glutamic acid levels in the HD group were significantly higher compared with those in other groups. Electronic tongue analysis revealed that sourness of the HD group was lower than that of other groups, whereas the HD group showed significantly higher umami, richness, and saltiness compared to other groups (p<0.05). Sensory test results revealed that the HW group had significantly higher tenderness, while the HD group had significantly higher chewiness, juiciness, and overall acceptability scores.

**Conclusion:**

These results suggest that both wet- and dry-aging treatments can effectively improve sensory characteristics, and dry-aging was much more useful to enhance umami tastes and meat quality of 2 grade Hanwoo sirloins.

## INTRODUCTION

Aging is the process of storage at low temperatures ranging from −1°C to 1°C to improve tenderness and flavor attributes of meat, resulting in meat quality improvements. Dry-aging is a traditional aging method, in which meat is kept in a ventilated and refrigerated room where available airflow, temperature, and relative humidity may be manipulated. Wet-aging is a method in which the meat is sealed in a plastic vacuum packing material and stored under refrigerated conditions. Several studies have demonstrated that dry-aging enhances the flavor of meat compared with wet-aging [1–3]. By contrast, some research has shown that slightly significant differences in consumer palatability attributes were found despite higher shrinkage rates and weight/trim loss in dry-aged beef [4,5].

The sensory properties of meat are influenced by the fat and protein contents in meat, which provide different qualities of tenderness and flavor. The sensory perceptions of meat are regarded as i) *antemortem* attributes such as species (pork, beef, and poultry), breed, and sex [6], and ii) *postmortem* attributes such as aging, packaging, and cooking methods [7–9]. The basic consumer sensory perceptions for meat have been described as aroma, flavor, tenderness, and juiciness [1,2]. Consumer sensory evaluation is an important process to determine meat quality but is also easily influenced by individual demographics and background characteristics [3] which can interrupt consistent data collection.

Recently, there has been a growing interest in electronic systems to evaluate food quality [10]. Previous research has taken advantage of electronic systems to analyze meat characteristics such as detection of nitrite and chloride contents in minced meat [11] and evaluation of fresh pork [12] and beef [13] meat quality. It is possible to evaluate the sensory characteristics of aged beef using an electronic system to eliminate the panel variations of the sensory evaluation.

Although many previous studies have researched the effects of aging methods on meats, controversy remains as to whether the increase in the taste of meat is detectable by consumers. In addition, there is limited information regarding the comparison among physicochemical and sensory characteristics using panels and taste profiles, which is confirmed by electronic tongue analysis of frozen and aged beef. According to our previous study [14], the deterioration of wet-aged beef quality after 40 days was detectable due to anaerobic microbial growth. Therefore, the objective of this study was to compare the effects of 40 days of dry-aging and 21 days of wet-aging on panel sensory characteristics and taste profiles using an electronic tongue instrument for beef. To ensure the benefits of aging were completed, commercial beef (non-aged beef) and frozen beef were prepared for these comparisons.

## MATERIALS AND METHODS

### Preparation of products

Grade 2 sirloins from six Hanwoo steers (about 30 months old) were obtained on day 5 after slaughter, from Livestock Products Nong-hyup Agricultural Cooperative Federation (Eumsung, Korea). After removing excessive fat and connective tissues from the sample surface, each muscle was cut into four sections, resulting in four samples per animal. A 1 kg Hanwoo sirloin from each treatment with a thickness of 10 cm was stored for 40 days of dry aging and 21 days of wet aging. Samples before storage were used as the control group (CON). There were four experimental groups: frozen+vacuum packed (Hanwoo frozen, HF) in a −18°C freezer; Hanwoo wet-aging (HW), vacuum packaged meat; Hanwoo dry-aging (HD), meat without packaging stored in a 1°C cooler (80% to 85% relative humidity and 0.2 to 0.3 m/s airflow). Samples requiring packing were stored in a vacuum packaged bag (Cryovac barrier bags, oxygen transmission rate 3 to 6 cm^3^/m^2^/24 h) for storage.

### Proximate composition

Proximate compositions (crude protein, crude fat, moisture, and ash) were measured according to the method of the Association of Official Agricultural Chemists [15]. Crude protein content was analyzed according to the Kjeldahl method. Crude fat content was determined using the Soxhlet extraction method. Moisture content was determined after drying the sample at 105°C overnight to a constant weight. Crude ash content was measured after burning samples at 550°C.

### Cooking loss

Samples were consistently cut to a 2.54 cm thickness (weight of samples, 50±5 g), which was placed in a commercial plastic bag. Each sample was heated in an 80°C shaking water bath (Diamond M, Julabo, Germany) until the core temperature reached 70°C as recorded by insertion of a waterproof thermometer (Testo Inc., Lenzkirch, Germany). The sample was then cooled to 4°C. Afterward, moisture on the surface of samples was removed. Cooking loss was calculated as the difference between the weight before cooking and after cooking using the following formula:

Cooking loss (%)=(Sample weight before cooking-sample weight after cooking)/sample weight before cooking×100

### pH

Using a Bag mixer 400 (Interscience Co. Ltd., Saint Nom, France), 2 g of sample was homogenized for 60 s with 18 mL of distilled water. The suspension was then used for pH measurement with a pH meter (pH 900, Precisa Co, Diet Ikon, Switzerland).

### Expressible drip

The expressible drip was determined according to the method of Benjakul et al [16]. Briefly, 0.3 g of sample was pressed through an expressible machine for 30 s with 10 J (9.9 kg/cm^2^) of force (IF 32B-S50, Ilshin Tech. Co. Ltd., Ulsan, Korea). The weight before and after pressing was compared and measured. The expressible drip was calculated using the following formula:

Expressible drip (%)=(Sample weight before pressing-sample weight after pressing)/sample weight before pressing×100

### Instrumental color measurement

Samples were cut for exposure to oxygen and placed at room temperature for 30 min. A chromameter (CR-400, Konica Minolta, Inc., Osaka, Japan) was used to measure the color of the surface of the sample. The machine was calibrated with a white plate (Commission Internationale de l’Eclairage [CIE] L* = +92.91, a* = −0.69, b* = +3.30). Measurements for CIE L* (lightness), CIE a* (redness), and CIE b* (yellowness) were repeated three times.

### Shear force

Samples used in cooking loss measurement were subjected to shear force according to the method of Wheeler et al [17]. After cooking loss measurements, the samples were prepared as six 1.27 mm diameter cores per each sample. The shear force was measured by positioning the sample horizontally and using a bladelike probe (TA-SBA, Brookfield Inc., Stoughton, MA, USA). Target speed was 3.00 mm/s, and the collected data was expressed as kg.

### Fatty acid composition

Fatty acid composition analysis of sirloin fat was performed according to Coorey et al [18]. Briefly, 25 mg of samples were added into a glass tube with 1.5 mL of NaOH/MeOH (NaOH 2 g+methanol 100 mL) solution. The glass tube with the sample was heated in a 100°C-heating block for 5 min. The sample was then cooled followed by the addition of 2 mL of 14% Boron trifluoride-methanol solution (125 g BF3/L MeOH) followed by heating at 100°C for 30 min. After cooling to 30°C, 1 mL of isooctane and 5 mL of potassium carbonate were added, and the solution was centrifuged for 10 min. The supernatant was extracted and analyzed by gas chromatography (GC 5890, Agilent Tech, Santa Clara, CA, USA) equipped with an SP-2560 column (100 m×0.25 mm×0.2 μm).

### Free amino acid contents

The free amino acid contents were determined according to the analytical method of Deniz et al [19]. Briefly, samples were freeze-dried, ground, and added to 0.01 N HCl. They were ultrasonically extracted for 1 h followed by extraction at room temperature for 24 h. Then, the supernatant was filtered with a 0.2 μm filter and then deproteinized by the addition of 3 volumes of 70% ethanol. Free amino acid contents were analyzed with an Ultimate 3000 HPLC system (DIONEX, Sunnyvale, CA, USA).

### Electronic tongue measurement

Water-soluble precursors were extracted using the method of Koutsidis et al [20]. Briefly, 3 g of the sample was placed in 10 mL of cold deionized water inside of a 50 mL tube and shaken for 5 min. After that, the tube was centrifuged at 10,000 ×g for 10 min at 4°C. The supernatant with the pellet was collected and added to a 50 mL tube. It was re-extracted with 5 mL of cold water. A Whatman No. 1 (11 μm) filter paper was used to remove impurities such as tissues and fats from the sample. The extracts were stored at −80°C until further analysis. The following standard compounds were prepared: HCl (sourness), NaCl (saltiness), MSG (umami), tannic acid (astringency), and MgSO_4_ (bitterness) at the same concentrations of 0.01 mol/L to check the cross-selectivity of the sensors. The electronic tongue analysis was performed using an E-tongue (Taste sensing system SA 402B, Insent Intelligent Sensor Technology Inc., Kanagawa, Japan).

### Sensory panel test

The sensory test was performed using 20 sensory panels (a total of 20 panels, 12 women and eight men aged 23 to 29 years with an average age of 26.2 years) from the Laboratory of Meat Science. All panels had basic knowledge and experiences in analyzing meat quality. According to Fabre et al [9], the samples were cooked on hot plates heated to 220°C on an electric grill until the core temperature of the samples reached 71°C. The cooked samples were then cut to 1 cm in thickness, 4 cm in length and 2.5 cm in width. The samples were served with randomized three-digit numbers. After eating an individual sample, the panel members rinsed their mouths with water and ate the next sample after waiting 1 to 2 min for the evaluation. The following five factors were evaluated using a 7-point hedonic scale for the tasting: tenderness (1, extremely tough; 7, very tender), chewiness (1, extremely elastic; 7, very easy swallowing), juiciness (1, extremely dry; 7, very juicy), flavor (1, extremely weak flavor; 7, very strong flavor) and overall acceptance (1, extremely dislike; 7 extremely like).

### Statistical analysis

The experimental design was a randomized complete block design with six replicates. The number of animals was blocked (n = 6). Each loin per animal was divided into four sections and randomly assigned to four treatments (commercial beef, frozen beef, 21 days wet-aged beef, and 40 days dry-aged beef). Averages and standard deviations of the test results with 95% confidence intervals were calculated. A one-way analysis of variance was conducted using an aging method as a fixed effect calculated using SPSS 24.0 (SPSS, Inc., Chicago, IL, USA; 1988). Tukey’s test was performed to compare significant differences (p<0.05).

## RESULTS AND DISCUSSION

### Proximate analysis, cooking loss, expressible drip, and pH

Results of proximate components and physicochemical characteristics in Hanwoo sirloin after the freezing or aging process are shown in Table 1. Moisture contents in all groups were significantly (p<0.05) reduced after 40 days of dry-aging. The HD group had the lowest moisture content at 54.5%. The fat content of the HD group was 17.5%, which was significantly (p<0.05) higher than that of the other groups. The HW group had a fat content of 2.6%, which was significantly (p<0.05) lower than that of other groups. The HF group had numerically (p<0.05) lower ash content compared with other groups. However, there was no significant (p>0.05) difference in ash content among treatment groups. Dry-aging was performed in a well-ventilated environment with humidity of 70% to 85% and a temperature of 0°C to 3°C. These conditions could result in a significant reduction in moisture due to evaporation [4,21].

The HF group had a significant increase in cooking loss compared with the other groups (p<0.05). This finding is in agreement with those of Shanks et al [22], who reported that ice crystal formation leads to increased cooking loss in frozen meat. HD had the lowest cooking loss among the groups (p< 0.05) and had significantly (p<0.05) lower expressible drip than other groups. The HW group had a significantly (p<0.05) higher expressible drip than the other groups. In the case of HD, fat content was higher, whereas the expressible drip of HD was significantly lower, compared to other groups. Moisture evaporated during dry-aging, resulting in increased protein and fat contents of HD compared with HW. Therefore, higher expressible loss and cooking loss resulted from the moisture loss by dry aging.

The pH of CON, HF, HW, and HD was shown to be 5.39, 5.52, 4.95, and 5.45, respectively. The pH of the HF group was significantly higher than that of the other groups, a result similar to that of Fan et al [23]. The HW group had the lowest pH value compared with the other groups. Li et al [1] reported that the number of lactic acid bacteria (LAB) increased when vacuum-sealed beef was stored for 14 days due to the anaerobic environment. In this study, the increase of LAB by vacuum packing might have produced lactate, thus decreasing the pH value.

### Color values and shear forces

Color values (lightness, redness, and yellowness) of the Hanwoo sirloin surface during the freezing or aging periods are shown in Table 2. Lightness and yellowness values were 46.91 and 14.56, respectively, in the HW group, which were significantly higher than those of other groups. The lightness and yellowness values in the HD group were 34.6 and 9.3, respectively, which were lower than those in other groups (p<0.05). The redness of HF (a*, 23.05) was lower than that of the CON (a*, 26.36). In addition, the dry- and wet-aged beef (a*, 22.05 and 19.07) showed a significantly lower redness than HF. According to Naveena et al [24], oxy-myoglobin content is decreased in refrigerated beef vacuum packed for 9 days, whereas met-myoglobin content is increased. Likewise, dry-aging reduces redness more than wet-aged beef, which was also reported by Kim et al [25].

The shear force of HF was higher than that of CON, whereas the shear force of HW and HD was lower than that of CON (p<0.05, Table 3). However, no significant difference was detected in the shear forces between wet-aged and dry-aged beef. Grouber et al [26] reported that tenderness is improved after aging beef sirloin for 28 days at 2°C. Kim et al [27] also indicated that an increase in tenderness by aging beef in a refrigerator is a result of the breakdown of the Z-disk in muscle myofibrils.

### Fatty acid compositions

The fatty acid composition of Hanwoo sirloins after the freezing or aging periods is shown in Table 3. The HW group had significantly lower 14:1n-5 and 16:1n-7 fatty acid percentages than the CON group, which had the highest monounsaturated fatty acids (MUFA) compared with the HD group. The percentage of 14:1n-5 and 16:1n-7 fatty acids in HF decreased compared with the other groups. In addition, HF showed the lowest polyunsaturated fatty acids (PUFA) among the groups (p<0.05). This result could be explained by the remaining lipolytic enzyme activity of meat during frozen storage in accordance with the findings of Alonso et al [28]. There was no significant (p>0.05) difference in saturated fatty acids among the groups. The HD group had higher 20:5n-3 fatty acid content compared with the other groups (p<0.05). Similarly, Kim et al [29] demonstrated that the C20:2 and C20:3n-6 content of beef increased during the aging process. However, less information is available on the change of fatty acids in meat during the aging process. Therefore, further study is necessary to determine the mechanism for increasing PUFAs during the aging process.

### Free amino acid contents

Free amino acid contents of Hanwoo sirloins after the freezing or aging periods are summarized in Table 4. The HF group showed lower contents of Asn, Asp, Ser, Arg, Tau, Val, Met, Phe, Iso, Leu, and Lys than the CON group (p<0.05). The HW group had significantly higher Asn, Ser, Phe, and Leu contents compared to other groups. The HD group had significantly (p<0.05) higher Glu, His, Gly, Thr, gamma-aminobutyric acid, Val, Try, and Lys contents compared with other groups. The aged beef groups (HW and HD) had significantly (p<0.05) higher Asp, Met, and Iso contents compared with the HF group. However, there was no significant (p>0.05) difference in Pro content among groups. Our results were in agreement with the results of Koutsidis et al [20] that Gly, Thr, Met, and Iso contents were increased if the aging of beef progressed in a refrigerator. In particular, the aspartic and glutamic acids increased in the aged beef (HW and HD) compared with CON and HF, whereas the aspartic acid did not show a significant difference between HW and HD. The glutamic acid content of HD was significantly higher than that of HW in agreement with Oh et al [30]. Meat protein degradation through endopeptidase action during aging storage, resulting in the increasing free amino acid contents, has been reported by previous studies [29,31]. Freezing seemed to not affect the breakdown of protein during storage significantly. Kristensen et al [32] demonstrated that calpastatin is activated regardless of the freezing state and that calpain was inactivated during freezing storage.

### Electronic tongue analysis

**Figure 1:**
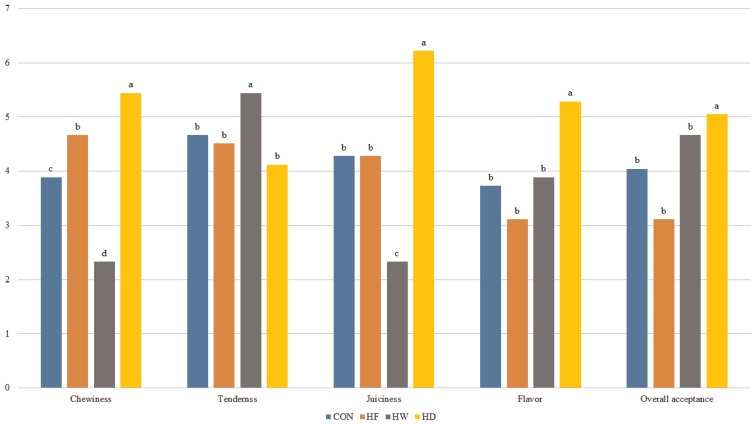
Electronic tongue scores of Hanwoo sirloins after the freezing or aging processes. CON, control group; HF, Hanwoo frozen; HW, Hanwoo wet-aging; HD, Hanwoo dry-aging. ^a–d^ Means sharing different letters in the same row are significantly different (p<0.05).

The results of electronic tongue analysis of Hanwoo sirloins after the freezing or aging periods are shown in Figure 1. The electronic tongue system is composed of 5 sensor probes that detect five particular tastes (sourness, bitterness, acerbity, umami, and saltiness) and two reference probes for greasy taste. The HD group had a lower sourness value compared with the CON group (p<0.05). For bitterness values, the HD group had the highest value, whereas the HF group had the lowest value (p<0.05). The HD group had the highest saltiness value, whereas the HW group had the lowest saltiness value compared with the other groups (both p<0.05). The HD group had values of 10.2 for umami and 2.1 for richness, which were significantly (p<0.05) higher than those for the other groups. It is known that free amino acid contents in beef have strong relevance to several tastes such as sweet, sour, bitter, and umami depending on the beef types. It has been shown that umami-taste free amino acids (aspartic acid and glutamic acid) with 5′-nucleotides can make umami [10]. Similarly, Kim et al [29] indicated that umami and saltiness in dry-aged round and shank muscles increased compared to wet-aged beef according to the results of electronic tongue analysis.

### Sensory test

**Figure 2:**
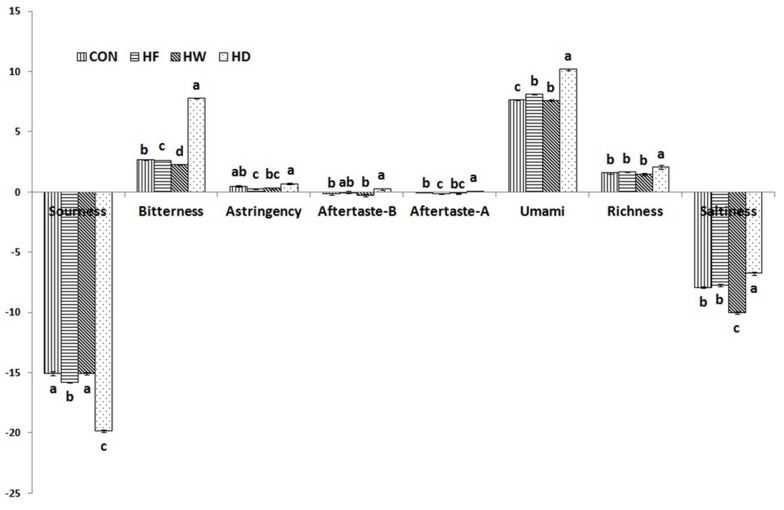
Sensory properties of Hanwoo sirloins after the freezing or aging processes, 7-Point hedonic scale (1 = extremely dislike, 7 = extremely like). CON, control group; HF, Hanwoo frozen; HW, Hanwoo wet-aging; HD, Hanwoo dry-aging. ^a–d^ Means sharing different letters in the same row are significantly different (p<0.05).

Results of sensory characteristics of Hanwoo sirloins after the freezing or aging periods are shown in Figure 2. For tenderness values, the HW group was significantly higher than the other group. There was no significant difference in tenderness values among CON, HD, and HF groups. The chewiness of the HD group was higher than that of the CON group (p< 0.05). The HD group had the highest juiciness score, whereas the HW group had the lowest juiciness and chewiness scores (p<0.05). There were no significant (p>0.05) differences in juiciness scores between the HF and CON groups. The HD group had a high juiciness score despite having the lowest water content. According to research by Campbell et al [33], dry-aged beef has higher tenderness and juiciness scores in the sensory test compared with fresh meat. The HD group had a significantly higher flavor score than the other groups. Warren and Kastner [21] reported that roasted flavor intensity of dry-aged beef increased compared with wet-aged beef, in agreement with Kim et al [25]. The significantly higher PUFA content in HD might have negatively contributed to its flavor score in this study. Dry-aged beef showed the highest scores for overall preference compared with the other groups.

## CONCLUSION

The dry-aging process improved the physicochemical, textural, and sensory characteristics of grade 2 Hanwoo sirloins. The cooking loss and expressible drip decreased in dry-aged beef due to the natural evaporation of moisture in dry-aged beef. The glutamic acid content related to umami taste of dry-aged beef increased, resulting in increasing umami and richness compared with non-aged, frozen, and wet-aged beef, and these findings were confirmed by the electronic tongue analysis. In addition, PUFAs increased in dry-aged beef. In terms of sensory evaluation using electronic tongue analysis and the panel test, the dry-aging process could produce meat with improved flavor and texture characteristics. However, it is necessary to prove the relationship among consumer sensory evaluations, descriptive sensory tests, taste precursors, and electronic tongue analysis of aged meat according to various aging methods and times in further studies.

## Figures and Tables

**Table 1:** Proximate analysis, cooking loss, pH, and expressible drip of Hanwoo sirloins after freezing or aging processes

Proximate analysis (%)	CON	HF	HW	HD	SEM	p-value
Moisture	70.25^a^	64.37^c^	67.58^b^	54.50^d^	0.62	<0.001
Protein	23.15^bc^	22.73^c^	23.70^b^	24.83^a^	0.26	<0.001
Fat	3.78^c^	8.93^b^	2.57^c^	17.47^a^	0.43	<0.001
Ash	1.07	0.98	1.03	1.04	0.03	0.07
Cooking loss	24.40^b^	26.46^a^	25.28^ab^	19.24^d^	0.49	<0.001
Expressible drip	39.08^b^	44.62^a^	33.08^c^	25.18^d^	0.78	<0.001
pH	5.39^c^	5.52^a^	4.95^d^	5.45^b^	0.02	<0.001

CON, control group; HF, Hanwoo frozen; HW, Hanwoo wet-aging; HD, Hanwoo dry-aging; SEM, standard error of the mean.

a–d Means sharing different letters in the same row are significantly different (p<0.05).

**Table 2:** Color values and shear forces of Hanwoo sirloins after the freezing or aging processes

Items	CON	HF	HW	HD	SEM	p-value
CIE L*	41.08^c^	42.15^b^	46.91^a^	34.60^d^	0.29	<0.001
CIE a*	26.36^a^	23.85^b^	22.05^c^	19.07^d^	0.17	<0.001
CIE b*	11.34^c^	13.13^b^	14.56^a^	9.25^d^	0.19	<0.001
Shear force (kg)	2.87^b^	3.44^a^	2.15^c^	2.52^c^	0.06	<0.001

CON, control group; HF, Hanwoo frozen; HW, Hanwoo wet-aging; HD, Hanwoo dry-aging; SEM, standard error of the mean; CIE, Commission Internationale de l’Eclairage.

a–d Means sharing different letters in the same row are significantly different (p<0.05).

**Table 3:** Fatty acid composition of Hanwoo sirloins after the freezing or aging processes (unit: g/100 g)

Items	CON	HF	HW	HD	SEM	p-value
C14:0	2.70	2.21	2.36	2.39	0.07	0.057
C16:0	24.1	18.23	19.31	18.74	1.01	0.088
C18:0	8.18	5.19	7.83	6.69	0.22	0.310
C20:0	1.02	1.70	2.66	3.05	0.46	0.482
C14:1n-5	1.44^a^	0.94^bc^	0.83^c^	1.04^b^	0.09	0.001
C16:1n-7	4.82^a^	3.27^b^	2.58^b^	3.56^ab^	0.02	0.020
C18:1n-9	42.99	35.47	33.78	31.67	1.83	0.061
C20:1n-9	1.99	4.15	6.46	4.87	0.78	0.247
C18:2n-6	5.28	2.89	4.59	4.83	0.41	0.148
C20:2n-6	0.74	0.27	0.79	1.09	0.13	0.139
C20:3n-6	0.27	0.42	0.52	0.48	0.06	0.494
C18:3n-3	3.82	2.19	3.74	5.01	0.54	0.394
C20:3n-3	0.31	0.26	0.51	0.34	0.04	0.248
C20:5n-3	0.64^b^	0.01^c^	0.01^c^	1.29^a^	0.21	0.005
SFA	35.99	27.32	32.15	30.87	1.41	0.159
MUFA	51.23^a^	43.81^ab^	42.98^ab^	41.14^b^	1.58	0.040
PUFA	11.74^a^	6. 67^b^	11.43^a^	10.40^a^	0.96	0.021
PUFA/SFA	0.20	0.39	0.36	0.38	0.04	0.236
w3	3,98	4.12	5.53	5.34	0.57	0.470
w6	3.58	3.80	5.90	6.39	0.82	0.628
w6/w3	1.77	0.90	1.07	1.27	0.03	0.790

CON, control group; HF, Hanwoo frozen; HW, Hanwoo wet-aging; HD, Hanwoo dry-aging; SEM, standard error of the mean; SFA, saturated fatty acids; MUFA, monounsaturated fatty acids; PUFA, polyunsaturated fatty acids; w3, omega-3 fatty acids; w6, omega-6 fatty acids.

a–d Means sharing different letters in the same row are significantly different (p<0.05).

**Table 4:** Free amino acid contents of Hanwoo sirloins after freezing and aging processes (unit: g/100 g)

Items	CON	HF	HW	HD	SEM	p-value
Aspartic acid	0.89^b^	0.60^b^	2.04^a^	1.83^a^	0.04	<0.001
Glutamic acid	5.96^c^	5.40^c^	8.48^b^	13.48^a^	0.46	<0.001
Asparagine	1.53^b^	1.22^c^	2.38^a^	0.83^d^	0.05	<0.001
Serine	3.10^c^	2.47^d^	4.11^a^	3.52^b^	0.09	<0.001
Histidine	1.65^b^	1.54^b^	1.61^b^	2.37^a^	0.07	<0.001
Glycine	2.86^b^	2.68^b^	3.35^ab^	3.55^a^	0.15	0.011
Threonine	3.21^bc^	3.02^c^	3.79^b^	4.68^a^	0.17	<0.001
Arginine	2.36^a^	1.33^b^	0.29^c^	0.66^bc^	0.16	<0.001
Alanine	12.73^ab^	16.23^a^	9.27^b^	10.46^b^	1.04	0.007
Taurine	27.68^a^	21.37^b^	20.99^bc^	19.29^c^	0.45	<0.001
GABA	0.05^b^	0.06^b^	0.10^b^	0.62^a^	0.01	<0.001
Tyrosine	0.29^NS^	0.21	0.56	0.74	0.97	0.006
Valine	3.19^c^	1.97^d^	4.46^b^	6.17^a^	0.19	<0.001
Methionine	1.90^b^	1.30^c^	3.41^a^	3.16^a^	0.10	<0.001
Tryptophan	0.50^b^	0.28^b^	0.41^b^	0.94^a^	0.09	<0.001
Phenylalanine	3.10^c^	2.37^d^	5.20^a^	4.55^b^	0.08	<0.001
Isoleucine	2.33^b^	1.43^c^	4.64^a^	4.50^a^	0.08	<0.001
Leucine	4.80^d^	3.15^c^	8.87^a^	8.17^b^	0.15	<0.001
Lysine	3.91^b^	2.54^c^	5.23^a^	5.83^a^	0.21	<0.001
Proline	1.24^NS^	1.45	1.19	1.98	0.18	0.052

CON, control group; HF, Hanwoo frozen; HW, Hanwoo wet-aging; HD, Hanwoo dry-aging; SEM, standard error of the mean; GABA, gamma-aminobutyric acid.

a–d Means sharing different letters in the same row are significantly different (p<0.05).
